# Supplementary material for: Causal Relationship between Aging and Anorexia Nervosa: A White-Matter-Microstructure-Mediated Mendelian Randomization Analysis
Source: Biomedicines. 2024 Aug 16;12(8):1874. doi: 10.3390/biomedicines12081874 (PMC11351342; doi:10.3390/biomedicines12081874)
Supplement: Supplementary file 1 [file biomedicines-12-01874-s001.zip › Supplementary Figure/Figure S4/Singe SNP plot of MR analysis for TL to ubm-b-2081.pdf]

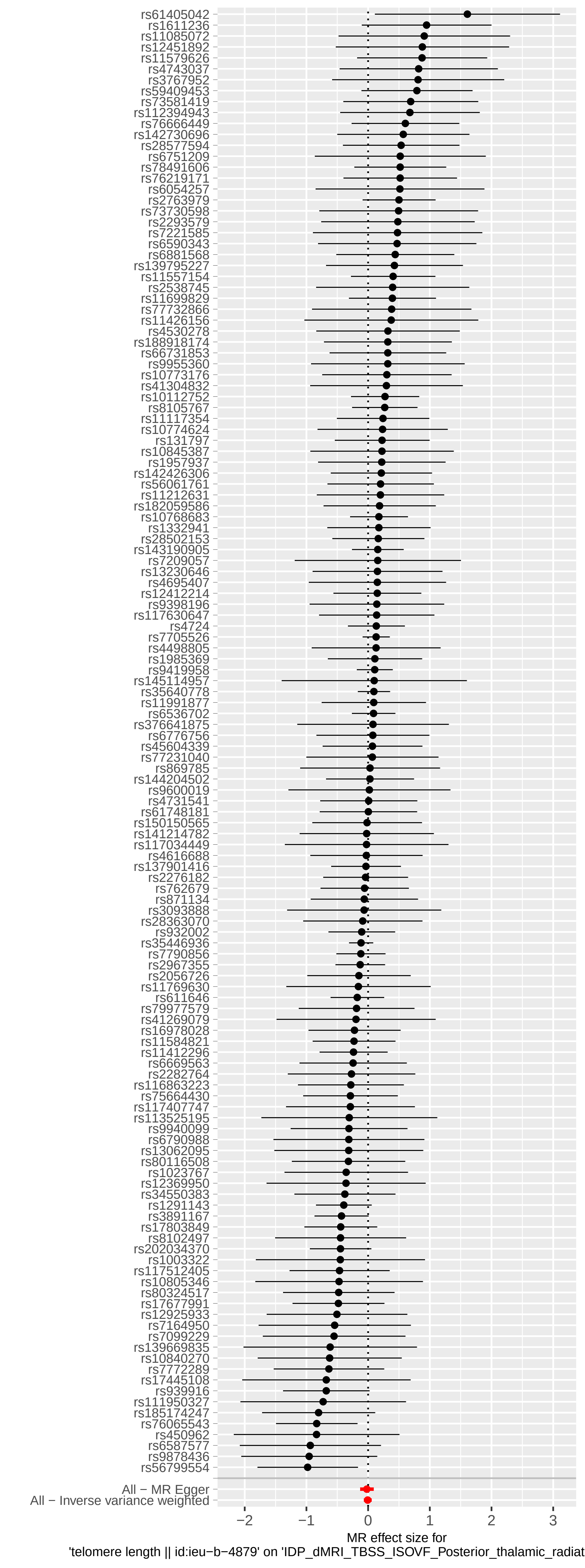
rs61405042
rs1611236
rs11085072
rs12451892
rs11579626
rs4743037
rs3767952
rs59409453
rs73581419
rs112394943
rs76666449
rs142730696
rs28577594
rs6751209
rs78491606
rs76219171
rs6054257
rs2763979
rs73730598
rs2293579
rs7221585
rs6590343
rs6881568
rs139795227
rs11557154
rs2538745
rs11699829
rs77732866
rs11426156
rs4530278
rs188918174
rs66731853
rs9955360
rs10773176
rs41304832
rs10112752
rs8105767
rs11117354
rs10774624
rs131797
rs10845387
rs1957937
rs142426306
rs56061761
rs11212631
rs182059586
rs10768683
rs1332941
rs28502153
rs143190905
rs7209057
rs13230646
rs4695407
rs12412214
rs9398196
rs117630647
rs4724
rs7705526
rs4498805
rs1985369
rs9419958
rs145114957
rs35640778
rs11991877
rs6536702
rs376641875
rs6776756
rs45604339
rs77231040
rs869785
rs144204502
rs9600019
rs4731541
rs61748181
rs150150565
rs141214782
rs117034449
rs4616688
rs137901416
rs2276182
rs762679
rs871134
rs3093888
rs28363070
rs932002
rs35446936
rs7790856
rs2967355
rs2056726
rs11769630
rs611646
rs79977579
rs41269079
rs16978028
rs11584821
rs11412296
rs6669563
rs2282764
rs116863223
rs75664430
rs117407747
rs113525195
rs9940099
rs6790988
rs13062095
rs80116508
rs1023767
rs12369950
rs34550383
rs1291143
rs3891167
rs17803849
rs8102497
rs202034370
rs1003322
rs117512405
rs10805346
rs80324517
rs17677991
rs12925933
rs7164950
rs7099229
rs139669835
rs10840270
rs7772289
rs17445108
rs939916
rs111950327
rs185174247
rs76065543
rs450962
rs6587577
rs9878436
rs56799554
All − MR Egger
All − Inverse variance weighted
−2
−1
0
1
2
3
MR effect size for
'telomere length || id:ieu−b−4879' on 'IDP_dMRI_TBSS_ISOVF_Posterior_thalamic_radiat
